# Tracing the urbanisation of risk in Malawi: A multilevel analysis

**DOI:** 10.4102/jamba.v16i1.1668

**Published:** 2024-08-15

**Authors:** Willi Bauer, Alexandra Titz, Mtafu C. Manda

**Affiliations:** 1 Institute of Geography, Faculty of Natural Sciences, Friedrich-Alexander-Universität Erlangen-Nürnberg, Erlangen, Germany; 2 Department of the Built Environment, Mzuzu University, Mzuzu, Malawi

**Keywords:** disaster risk reduction, urban vulnerability, governance, multilevel analysis, global south, Malawi

## Abstract

Cities in Malawi have long been outside the focus of disaster risk reduction. As a result, urban risks remain poorly understood, and urban governments and disaster risk reduction (DRR) practitioners working in cities struggle to adequately support vulnerable urban populations. This is evidenced by recent disasters such as Cyclone Freddy, which devastated the city of Blantyre in 2023, and increasingly common small-scale events in urban areas.

This article analyses the Malawian city as a distinct riskscape, shaped by national-level policies of neglect that create an institutional void that DRR practitioners working at the city level struggle to fill. This process is complicated by a multitude of challenges at different levels of governance, especially leaving small-scale events prevalent in urban areas largely unaddressed. This process of risk accumulation is increasingly affecting urban residents. Methodologically, we demonstrate this through a comprehensive policy analysis and by drawing on expert and civil society interviews and questionnaires conducted in Lilongwe City.

**Contribution:**

By outlining the interlocking challenges at multiple levels and grounding them in empirical data, we highlight the specificities of urban DRR efforts in Malawi and provide opportunities to improve them.

## Introduction

### Understanding risk accumulation in urban Malawi

Urban livelihoods in many cities are increasingly influenced by risks and uncertainties. As stated in the Sixth Assessment Report of the Intergovernmental Panel on Climate Change (IPCC), ‘in all cities and urban areas, the risk faced by people and assets associated with climate change has increased’ (Dodman et al. 2022:909). The report locates the steepest increase in urban vulnerabilities in smaller and medium-sized urban centres in low- and middle-income nations. Moreover, as explained by Maskrey and Lavell (2023:235), multidimensional poverty, the largely unplanned expansion of cities, and the access to basic services and infrastructure have in many places led to ‘large sections of the urban population being exposed to extensive and intense risks on a daily basis’. Yet, despite growing scholarly recognition (see Adelekan et al. 2015; Dodman et al. 2019; Fraser et al. 2017; Pelling & Wisner 2009), studies on urban risks and vulnerability in Africa, especially in sub-Saharan Africa, are still underrepresented. Furthermore, scholarly attention is mostly concentrated on capital or coastal cities (Cutter 2021). Small- and medium-sized inland cities are often overlooked. And while various actors are responding to these challenges and promoting urban disaster risk reduction (DRR), limited data availability and an incomplete understanding of the interplay between urbanisation and risk hinder their work (UN Habitat 2020).

Malawi is one of the world’s most vulnerable countries to climatic shocks (Warnatzsch & Reay 2019). However, public attention, as well as risk and vulnerability assessments have tended to focus only on rural areas (Chiusiwa 2015), as cities have been considered to be inherently more resilient. While this understanding has slowly started to change over the past decade, the devastation of the city of Blantyre by Cyclone Freddy in early 2023 served as a potentially paradigm-shifting demonstration of urban vulnerability. It also epitomises a persistent focus on infrequent, large-scale disasters. The public failure to consider cities and small-scale risks is also reflected in the scholarly literature, with few studies detailing vulnerabilities and risks in urban Malawi (for exceptions, see: Kita 2017b; Manda & Wanda 2017; Wanda et al. 2017). Consequently, the urban environments of risk accumulation and the vulnerabilities of urban residents remain poorly understood.

This article seeks to contribute to a better understanding of risk accumulation in Malawian cities as a multi-layered process shaped by urbanisation as well as by national policies hindering the work of DRR practitioners at the city level and permeating into urban living environments. This can be traced through the case of Lilongwe, as the city has only recently been designated as disaster-prone, mainly because of a sharp increase in localised flooding events (Makuwira 2022). Its analysis, thus allows us to better understand the recent uptick of urban risks, as well as the ramifications of the historical neglect of urban vulnerability for DRR practitioners seeking to address the growing challenges and the issues faced by urban residents in coping with the consequences. In doing so, we seek to raise awareness of specific urban risks and DRR governance challenges in order to stimulate further discussion and identify ways to strengthen DRR in cities. In what follows, we firstly present the context of urban development in Malawi, our conceptualisation of risk, and our methodology. Secondly, we detail recent disaster-related developments at the national level to facilitate an understanding of the governmental DRR structures – and their failure to consider cities as specific riskscapes. Thirdly, we move to the city level and analyse how DRR practitioners and municipal actors are trying to fill the institutional void created by rural-centric national policies. Fourthly, building on fieldwork conducted in Kawale, Lilongwe, we detail how these struggles spread into urban wards. Finally, we discuss the findings to highlight relevant areas for improving urban DRR in Malawi.

## Contextualisation and conceptual approach

### Urban development and challenges in Malawi

Malawi is still predominantly a rural country, with less than 20% of its population living in urban areas (National Statistical Office 2019:10). Its four main cities are Lilongwe, Blantyre, Zomba and Mzuzu. The population of Malawi has almost doubled since the beginning of the millennium, reaching about 20 million in 2022. Urban growth, driven by migration and high birth rates, has been even faster (see Table 1). Long seen as a negative trend, urbanisation is now recognised as a key area for development in ‘Malawi Vision 2063’, the country’s long-term development strategy (GoM 2020).

**TABLE 1:** Population of main urban centres.

City	1977	1987	1998	2008	2018
Lilongwe	98 718	223 318	440 471	669 532	989 318
Blantyre	219 011	333 120	502 053	648 852	800 776
Mzuzu	16 108	44 217	86 980	127 539	221 272
Zomba	24 234	43 250	65 917	81 501	105 013
Total	358 071	643 905	1 095 421	1 527 424	2 116 379
Population of Malawi	5 547 460	7 988 507	9 933 868	13 029 498	17 563 749
Share of four main cities (%)	6.45	8.06	11.03	11.72	12.05

Much of the urban population growth, especially since the advent of multiparty democracy in 1994, has taken place in informal settlements, as the demand for housing far outpaces the supply and urban planning, or at least its recognition and implementation (Chiweza 2019; UN Habitat 2023). For instance, an estimated 75% of the city of Lilongwe’s population lives in informal settlements (Lilongwe City Council [LCC] 2021). Beyond informality, extensive sprawl, insufficient infrastructural supply and land degradation are the major challenges.

Likewise, limited financial means to provide basic services is another main challenge faced by the government (Mangani 2022). Urban municipalities are particularly under-resourced. As part of the 1998 National Decentralisation Policy, the central government only provides a limited share of the municipal budget, forcing city authorities to generate revenue. In addition, land tenure conflicts, high rates of unemployment, poor law enforcement and limited institutional capacity are other major challenges faced by municipalities (Chiweza 2019). Losses from disasters are also having an increasing impact on public and municipal budgets.

### Conceptualising risk and disasters

Hazards may be natural, but disasters are not. Disasters are socially constructed products where historically rooted, pre-existing inequalities often render marginalised parts of the population disproportionately vulnerable (Adger 2006; Oliver-Smith 1999; Wisner et al. 2003). Critical disaster scholars emphasise that this condition of vulnerability and its social differentiation ‘is generated by social, economic and political processes that influence how hazards affect people in varying ways and different intensities’ (Wisner et al. 2003:7). It is increasingly recognised that the social, economic and political structures of the societies in which people live are the main causes of people’s vulnerabilities (Bankoff & Hilhorst 2022; Collins et al. 2015). These structures have a significant impact on the ways in which individuals are unequally exposed and vulnerable to hazards, and hence the risk they face (Bankoff 2018; Wisner et al. 2003).

There are differing views on the meaning of ‘risk’. Geographical risk research, in which this study is embedded, aims to minimise risk and draws on concepts from the natural, social and cultural sciences. Risk is conceptualised as a function of hazards and vulnerability, whereas vulnerability is seen as the result of political, economic and social processes (see Wisner et al. 2003). Conversely, more recent social science approaches see risk as a prerequisite for securing livelihoods. They argue that individuals or groups must first translate hazards into manageable risks through understanding, negotiation and sensitisation. Risk, as the probability of possible outcomes of action, then enables the creation of security and the establishment of conditions for the reduction of social vulnerability (see Krüger & Macamo 2003; Müller-Mahn 2007).

In this article, we draw on the former conception of risk, as it aligns with *international* framings, such as the Sendai Framework (ed. UNISDR 2015), which influences Malawian DRR policies. We also employ the ‘risk spectrum’ (Bull-Kamanga et al. 2003; Dodman et al. 2019) as a useful tool for differentiation. Through it, disasters, small-scale disasters and everyday hazards are described as a continuum of risks marked by increasing frequency but decreasing singular effect. Disasters, such as Cyclone Freddy, result in widespread damage but are rather infrequent. Small-scale disasters, such as localised flooding are more frequent, but their effects are often limited, for example, to an urban ward. Everyday hazards, such as diseases caused by polluted drinking water, are an almost ubiquitous threat in many African cities that cumulatively affect a large number of persons. While disasters draw widespread attention, most fatalities in urban Africa are caused by small-scale disasters and everyday hazards. Addressing the full spectrum of risks can help to understand the relative importance of hazards, specific vulnerabilities, interactions between hazards and cascading effects, and the underlying drivers of risk (UN Habitat 2020). It thus provides a way of moving beyond ‘disaster talk’ to ‘risk reduction talk’ (Bull-Kamanga et al. 2003:200).

## Research methods and design

**FIGURE 1:**
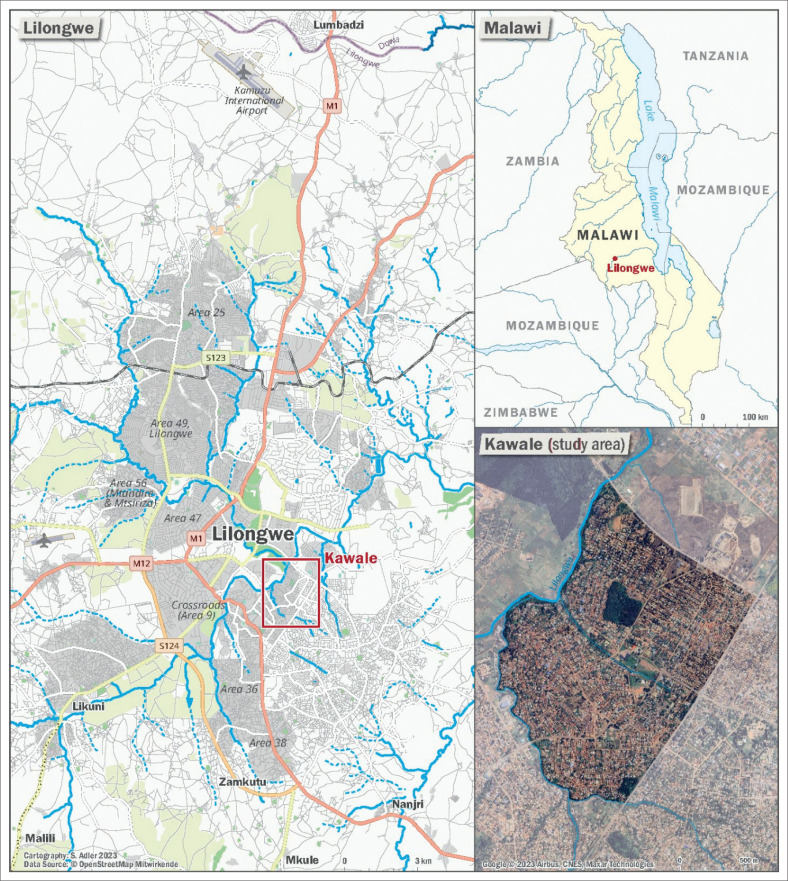
Location of Kawale (Area 7), Lilongwe.

The study was conducted in Lilongwe, Malawi’s capital. Lilongwe has only recently been designated as a high-risk area and is barely considered in the generally limited DRR-related research on Malawian cities (for exceptions, *see* Makuwira 2022; Mwalwimba, Manda & Ngongondo 2023). Specifically, research was conducted in Kawale, a high-density, low-income area bordering on the Lilongwe River (see Figure 1).

To trace the urbanisation of risk across national, city and ward levels, this article draws on several methods. Firstly, relevant national policy and legal documents were analysed to understand the embedding of DRR at the national level. This analysis was also used to reflect on changes in the conceptualisation of disaster and risk, and to identify relevant stakeholders. Secondly, qualitative interviews were conducted with DRR professionals from both the government and leading non-governmental organisations (NGOs). Initial interviewees were selected based on the previous document analysis. Further interview partners were contacted using snowball sampling. A total of 16 narrative interviews, including seven group interviews, were conducted and ranged in length from 35 to 160 min. Where consent was given, the interviews were recorded and transcribed. Notes were taken during all interviews. Thirdly, qualitative and quantitative methods were employed to unpack local perspectives on risk in Kawale. The two chiefs of Kawale were interviewed individually by the lead author and a Chichewa-speaking field assistant. The block leaders, elected ward representatives, and the two ward councillors representing Kawale at the municipal level were invited to a joint group discussion. A total of 10 representatives participated in the discussion. In addition, 29 guided civil society interviews were conducted to include local perspectives. The interviews were conducted in Chichewa or English. Interviewees were purposively selected, with a focus on adult residents to draw broadly on experiences and learn about changing risk patterns. Finally, 101 exploratory semi-structured questionnaires were completed by local residents, all of whom verbally gave their informed consent. The sampling was purposive to ensure that all spatial parts of Kawale were equally represented. Therefore, the study does not claim to be representative.

The notes and transcripts of all qualitative interviews were analysed for core topics and interrelated themes. This was performed using *Obsidian*, a knowledge management tool that illustrates links between codes and text. *Obsidian* allowed the document analyses to be linked, allowing the uptake of national-level frameworks to be traced across scales, while being sensitive to inconsistencies and local needs not met by overarching policies (or DRR practitioners). To allow for a coherent multilevel analysis, the following sections briefly illustrate the institutional embedding of DRR in Malawi and sensitise for recent developments.

## State of disaster risk reduction in Malawi: Institutional setup and context

### The institutional setup of disaster risk reduction in Malawi

**FIGURE 2:**
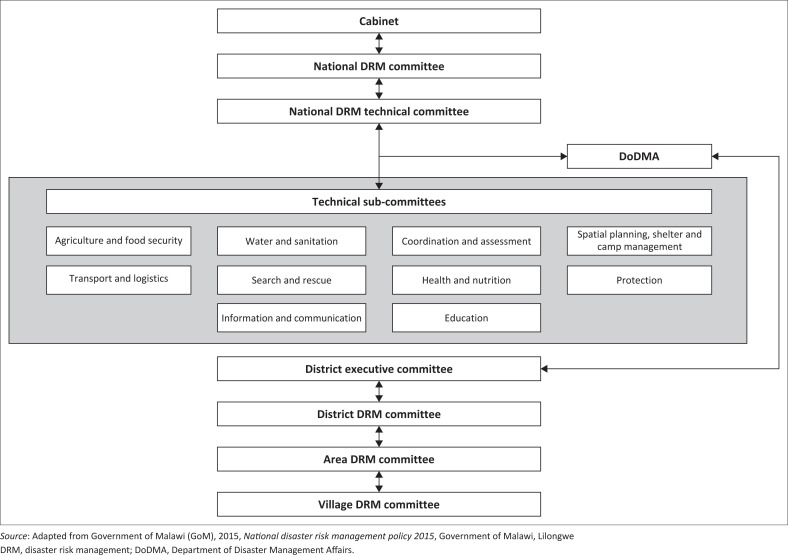
National disaster risk reduction structure after Government of Malawi 2015.

Disasters are part of Malawi’s history. In 1946, for instance, the colonial capital Zomba was devastated by landslides (Edwards 1948). However, disasters and relief action were only legally defined in 1991. The *Disaster Preparedness and Relief Act* (GoM 1991) provided the legal guideline for disaster response until the recently enacted Disaster Risk Management Bill (GoM 2023). The former also established the Department of Disaster Management Affairs (DoDMA). The two legal documents are supported by policy frameworks. Most important of these is the 2015 National Disaster Risk Management Policy (GoM 2015). It outlines the institutional set-up for DRR (see Figure 2), which is partly decentralised. The ‘National DRM Technical Committee’, made up of various public and non-governmental stakeholders, and the DoDMA link the central government to the districts, which are tasked with implementation efforts. The national committee provides technical guidance on both implementation and policy formulation for DRR. The DoDMA’s mandate is to guide and coordinate DRR implementation efforts.

Disaster risk reduction is also included as a development area in the Malawi Growth and Development Strategy (2017–2022) (GoM 2017). However, it is barely mentioned in its successor, the Malawi Vision 2063. Pardoe et al. (2020) also observe a decreasing momentum regarding climate change adaptation. With little long-term consideration, national DRR policies and efforts remain closely tied to disasters.

In addition to the statutory institutional setup, DRR in Malawi is heavily donor-driven. In a regional comparison of 13 African countries, Malawi’s public DRR budget allocation percentage ranks among the lowest (Tiepolo & Braccio 2020). Third-party funding is a necessity for addressing existing financial impasses; however, heavy donor reliance challenges institutional set-up while also influencing policy formulation and implementation (Kita 2017a). For example, the 2015 policy was developed after international donors tied monetary support for flood relief to the formulation of a national DRR policy (Leck et al. 2018:7). To limit overt external influence and to better understand local disaster cultures and vulnerabilities, Hendriks and Boersma (2019) thus call to ‘bring the state back in’.

### Hazards and recent disasters in Malawi

**FIGURE 3:**
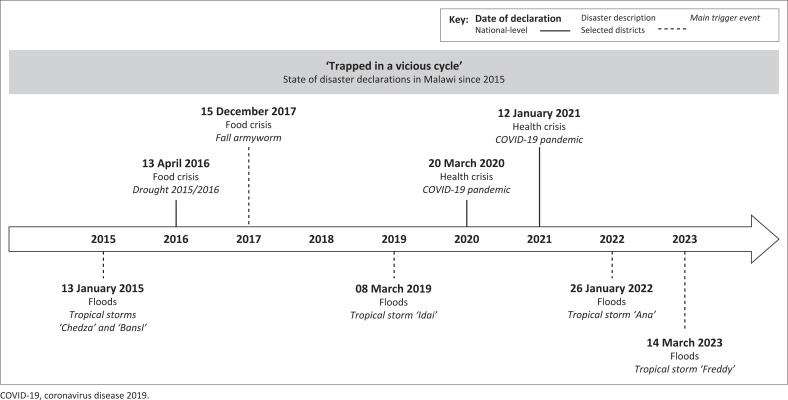
Presidential state of disaster declarations in Malawi since 2015.

Malawi has been caught in a ‘vicious cycle of getting hit again while trying to get up’ (DoDMA 2023). In the years from 2015 to 2023, eight individual states of disaster have been declared (see Figure 3). A state of disaster can be declared in the case of an emergency that mandates extraordinary measures and exceeds institutional capacities (GoM 2023:20). The declaration can be a powerful tool to unlock additional funding and allow for (external) humanitarian interventions. However, it is a highly political issue and often delayed or avoided to not create the appearance of governmental weakness.

On four occasions, tropical storms and subsequent flooding necessitated the declaration of state of disaster. In 2016, then-President Peter Mutharika declared a national state of disaster because of a prolonged dry spell, exacerbating a severe food crisis caused by the previous years’ floods. Historically the most prevalent risks in Malawi, floods and droughts, have recently increased in frequency and impact (GoM 2022). Furthermore, diseases, viruses and pests are a frequent cause of premature death and loss of livelihood. In 2017, the fall armyworm caused crop losses, leading to a severe food crisis (GoM 2018). The *coronavirus disease 2019* (COVID-19) resulted in two emergency declarations by two presidents. Accordingly, the most recent multi-hazard contingency plan identifies floods, dry spells, storms, diseases and pests as the main hazards in Malawi (GoM 2022). In addition, the Northern Region of Malawi is partly susceptible to earthquakes (Manda & Wanda 2017), while lightning and strong winds pose smaller-scale hazards.

## The urbanisation of risk in Malawi

The urbanisation of risk is a process concurrent with the increase of large-scale disasters, characterised by a sharp increase in the frequency of small-scale events, such as localised flooding (Makuwira 2022:57). All Malawian cities, long considered to be comparatively safe, are now designated as vulnerable to floods and disasters in general (DoDMA 2023). However, the urbanisation of risk entails more than considering the city as a scale of reference for DRR planning. It is also composed and shaped by the city and the urban. The city is not only a specific site of risk accumulation, mitigation and governance; it is also a social lifeworld in which risks may be perceived, considered, valued and acted upon differently than elsewhere (Leck et al. 2018; Maskrey & Lavell 2023). In other words, cities are distinct ‘riskscapes’ (Müller-Mahn 2015; Müller-Mahn, Everts & Stephan 2018) that must be understood in order to facilitate effective and inclusive DRR.

While the following findings cannot provide comprehensive detail on local perceptions and valuations of risk, they do provide entry points for further research by illustrating the challenges of risk governance in urban areas and their conception by urban residents. Therefore, the following sections narrow in scale, starting with national policies framing the political and legal environment for the urbanisation of risk. The resulting challenges and constraints for municipal governments and DRR practitioners working at the city level are then detailed. Finally, local perspectives from Kawale are added to understand how the challenges of urban risk governance transfer to urban lives at risk.

### National level

Besides macroeconomic factors limiting budget allocation and capacity building while necessitating donor support, we identify two factors as crucial components of the urbanisation of risks on the national level: reactionary governance and urban neglect.

#### Policy-making through disasters, disaster-making through policies

Malawi’s disaster policies retell Malawian disaster history. The inaugural legal framework, the 1991 *Disaster Preparedness and Relief Act* (GoM 1991), was introduced after the Phalombe floods. The 2015 *National Disaster Risk Management Policy* (GoM 2015) was introduced after the floods and anticipated food shortage in 2015 and 2016. Similarly, the recently introduced *Disaster Risk Management Bill* (GoM 2023) was rapidly pushed through parliament and the courts after the devastation caused by Cyclone Freddy. Like the 2015 policy, the bill had originally been formulated much earlier. A 2019 draft was even approved, but not enacted in time. As a result, it had to go through the formal approval process again, where it was stuck until early 2023. In the wake of Cyclone Freddy, however, it took only a few weeks to amend, approve and enact the bill.

Disasters provide a window of opportunity for policy formulation and the adoption of legal frameworks. However, these legal frameworks and policy formulations transfer poorly into non-emergency situations and cohesive long-term planning for DRR. This is illustrated by the (non-)conception of risk in the two legal frameworks from 1991 to 2023. While titled *Preparedness and Relief Act*, neither risk nor hazard is mentioned in the Act of 1991, which clearly emphasises relief action. The 2023 DRM Bill updates and expands on the earlier act. However, it illustrates the political moment of the law’s acceptance. The Bill’s 2019 draft defined disaster risk as ‘the *potential* disaster losses, in lives, health status, livelihoods, assets and services, which *could* occur to a particular community or a society over some specified *future* time period’ (emphasis added, GoM 2019:7). This description is in line with the Sendai Framework (ed. UNISDR 2015), to which Malawian disaster policies are supposed to conform. In the enacted post-cyclone bill, the definition reads: ‘“disaster risk” means the potential disaster associated with loss of life, health status, livelihoods, assets and services, which may occur to a particular community over a period of time’ (GoM 2023:4). Risk is now defined through disaster, while previously contained definitions for disaster mitigation and disaster preparedness are omitted. This conceptualisation is, by default, reactive and continues a general prioritisation of post-disaster efforts. At last, the bill was passed quickly to demonstrate government action and decisiveness. Yet, with only 18 of 61 requested amendments included, the new act, while considered a step forward from the outdated legislation of 1991, has been criticised by many as being rushed (Interview, Malawi National Youth Network on Climate Change [NYNCC], 16 April 2023; Interview, UN Habitat, 18 April 2023).

Reactionary governance and limited long-term adaptation planning are a perpetual challenge for DRR in Malawi, which is also reflected in the work of non-governmental actors. As NGO representatives pointed out, ‘we complemented government which was reactive to disasters, so that was our approach as well’ (Group Interview, Habitat for Humanity, 05 May 2023). This, along with limited financial support from the government and limited autonomy for the DoDMA, severely undermines capacity building and mitigation. Furthermore, the retroactive focus on disasters and disaster *management* creates an institutional void that fosters the accumulation of risk. While this is not a challenge specific to urban areas, it is compounded by a scalar mismatch between disasters and cities, as well as traditional localisations of vulnerability.

#### Localising and governing disasters and vulnerabilities elsewhere

Cities and disasters have historically been considered as distinct. Even though this conception is changing, its institutional imprint still strongly influences urban DRR in Malawi. In general, DoDMA is tasked with guiding and coordinating efforts carried out in Malawi’s districts (GoM 2015:10). The delegation hierarchy on the sub-national level is from district disaster risk management (DRM) committee to area DRM committee to village DRM committee (see Figure 2). Cities are not considered separately. This can be traced back to a traditional anti-urban bias, with Malawian policy long viewing urbanisation as a negative process that must be countered by rural development (Brown 2011:942). This sentiment is prominently expressed in Malawi’s decentralisation policy. Municipal governments have limited legal rights and autonomy, as they fall under the jurisdiction of the district offices; this complicates the uptake of responsibilities by the City or District Council, respectively. Smaller towns, such as Karonga, rely entirely on the District Council – or remain ungoverned in terms of DRR (Manda 2014). Even Lilongwe City does not employ any designated DRR officers, creating a dependency on voluntarism and third-party support. The DoDMA has also traditionally focussed on rural areas and it has institutional staff and structures throughout the country, except in the four major cities (GoM 2022:25), with its national office not being intended *for* Lilongwe City, despite being located *in* it, on Capital Hill.

This structure also complicates the reporting of incidents. The legal procedure dictates that events within municipal boundaries must be reported to the (rural) District Council, which in turn delegates back to the city. However, as pointed out by Kita (2017a), most district offices are understaffed and do not feature a permanent DRR officer beyond junior level. This creates long communication chains, discouraging the reporting of small-scale incidents, while district officers often struggle to fulfil their mandate. City councils, on the other hand, have limited power to meet the expectations of urban residents as they don’t have designated capacities for DRR or relief efforts. Furthermore, data disaggregation for urban areas becomes challenging because of their collection at the district level (Leck et al. 2018:6–8). However, district-level risks may well vary from urban risks. For the Lilongwe district, for instance, strong winds are a key hazard, with floods playing a limited role, despite the prevalence of the latter in the city of Lilongwe (Interview, District DRR Officer, 05 December 2023). These mechanisms complicate a thorough understanding of urban risks. Especially everyday hazards and small-scale events tend to go unreported.

Beyond urban risks, urban vulnerability in Malawi is also poorly understood. Traditionally, cities have been excluded from vulnerability assessments, based on the assumption that they are resilient (Chiusiwa 2015). This notion is, for instance, reflected in the ‘Malawi Hazards and Vulnerability Atlas’ (DoDMA 2015), where all Malawian cities are depicted to be highly exposed to multiple hazards, yet their vulnerability is considered to be low to very low. This is partly explained by the generalising metrics employed, such as average household income. As a result, assessments may depict an image of overarching resilience while missing individual vulnerability. This links to the critique of one interviewee that:

‘[*A*]ll policies only mention community vulnerability, not individual groups.’ (Interview, NYNCC, 16 April 2023)

Yet, as argued by Titz et al. (2018), ‘community’ is an elusive concept that tends to homogenise its members, as well as their needs and demands, while neglecting tensions and differences. For example, the interviewed block leaders in Kawale pointed at sand mining as an illegal practice they want to limit; yet, one of the chiefs is a sand miner (Group Discussion, Kawale, 17 October 2022). Similarly, outsiders to the ‘community’ are at risk of being overlooked by vulnerability assessments, as well as being bereft of support by the ‘community’.

These issues make it difficult to fully understand the entire spectrum of risks in urban areas and the vulnerability of urban residents. They also have a negative impact on the work of practitioners and stakeholders involved in urban DRR. Based on expert interviews, the following section highlights these issues in order to provide a better understanding of risk governance at the city level in Malawi.

### City level: Governing the city-sized void of disaster risk reduction

Insufficient funding is a fundamental challenge for DRR governance in Malawi, regardless of region. In addition, the DRR practitioners interviewed outlined six main challenges to their work in urban areas:

- A lack of reliable, small-scale data, plus difficulties and challenges in data dissemination
- Limited uptake of risk-related information
- Weak law enforcement
- Limited long-term adoption of DRR measures
- Limited experience of working in urban areas
- A lack of caretaking by citizens.

Firstly, data availability is a general challenge for DRR in Malawi. Data collection on risks is still fairly new and often partial rather than comprehensive. Also, data processing is a challenge. For example, weather data are collected at the district level but is barely requested by District Councils, while the Department for Climate Change and Meteorological Services (DCCMS) itself struggles to process and disseminate data because of staff shortages (Interview, DCCMS, 20 July 2023). In the end, the data are generalised and shared only upon request. Likewise, early warning systems only reach parts of the relevant population, in certain areas, and even then, they provide little advance notice. The Lilongwe and Blantyre districts, home to Malawi’s two largest cities, are not yet covered (International Day of DRR, 13 October 2022). Also, the resolution of data are often incompatible with urban areas. Riverine flooding can only be forecasted at the catchment level and drainage floods remain a major unpredictable risk in urban areas. These challenges undermine the action potential of the forecasts.

Secondly and relatedly, there is a limited uptake of risk-related information. While distribution challenges are part of this, the historically low reliability of forecasts, despite major improvements, has damaged public trust in forecasts (Group Interview, DoDMA, 11 October 2022). Also, the formal disconnect between urban areas and risks seems to be mirrored in the perception of warnings. One interviewee described how residents in Blantyre did not seem to believe that warnings about Cyclone Freddy were directed at them (Interview, AEJ, 18 April 2023). This is confirmed by other actors:

‘Yes, it’s difficult to reach out to the rural areas, but they are the ones that are much more interested than the urban. I think maybe because there are a lot of interventions that go to the rural areas: they are informed, they have a lot more information […] than most of the urban people. So, whilst they are difficult to reach, they are also the ones who are knowledgeable when it comes to the issue of extreme events.’ (Interview, DCCMS, 20 July 2023)

Thirdly, a lack of enforcement of laws and by-laws in urban areas co-creates risk. In urban areas, the pressure on the legal system, for example, because of the demand of housing and developable land, is high. Yet, law enforcement is often lacking:

‘When you look at urban areas, many of the challenges that we are facing fall on the shoulders of enforcement of our laws and regulations. Because people are settling in areas that are not supposed to be settled [*in*] […]. People are just throwing solid waste everywhere. […] The issue of sand mining, the issue of agriculture along high-risk areas, we have got laws against all those. So, it’s different in rural areas where you have got natural processes that may be beyond our control. But when you talk of urban areas, you find that most of the challenges […] are man-made because we haven’t been able to properly regulate them.’ (Group Interview, DoDMA, 04 August 2022)

This is partly because of the disadvantaged position of cities in Malawi’s decentralisation process. Politics and corruption also play a major role (Makuwira 2022:62). For instance, high-income neighbourhoods in Lilongwe were flooded in 2020 as a result of ‘legal encroachments’ that violated the building plans but were officially approved (Interview, Department of Physical Planning, 05 July 2022). At the same time, the centralisation of police forces slows down enforcement processes, as city governments must formally request action in a lengthy process (Interview, NPC, 13 October 2022).

The fourth issue mentioned, the limited long-term success and uptake of DRR measures, is not specifically an urban problem (Dewa, Makoka & Ayo-Yusuf 2021), yet it interrelates with other urban DRR challenges. One public officer identified civic awareness as a key factor:

[*I*]f you go to the district and ask someone ‘What are disasters?’, the first response is ‘when someone was affected and DoDMA provided relief items’ and afterwards, they forget about it. (DoDMA 2023)

However, tokenistic participation commonly plays a role in incomplete mutual understanding. Information is often pushed to those affected or to civic leaders without mutual exchange. This, for instance, contradicts the called-for inclusion of local knowledge in DRR (Šakić Trogrlić et al. 2022). Furthermore, DRR in Malawi is very project-based and short-term because of its high donor dependency. Thus, results often do not meet the expectations of residents (Group Discussion, Kawale, 17 October 2022). Also, NGOs often do not provide an ‘exit strategy’ for the period after a project, which undermines the longevity of implemented measures while causing frustration (Šakić Trogrlić et al. 2018).

For urban areas, scepticism about the effectiveness of project-based DRR, along with the limited experience of DRR professionals working in cities are major challenges. As stated by NGO representatives:

‘[*O*]ur previous strategy had no focus on the city whatsoever.’ (Group Interview, Habitat for Humanity, 05 May 2023)

Given the relative newness of DRR-related work in cities, there is limited best practice to draw on. Cyclone Freddy painfully demonstrated the impact of limited data and experience, combined with changing climate patterns and urban development. Disaster warnings in Blantyre during the storm were confined to flooding, overlooking the potential for landslides (Interview, DCCMS, 20 July 2023). Caused by deforestation, hillside development and unprecedented rainfall, landslides were responsible for the majority of fatalities in the city of Blantyre.

Finally, governmental actors and NGOs criticise what they perceive as a lack of care for the city on the part of its inhabitants. This is partly explained by a limited attachment to the city (Interview, former LCC CEO, 26 April 2023). Likely reasons are newness to the city, close relations to home villages, and politics not being favourable to newcomers. Combined with a lack of law enforcement, actors also describe a feeling that ‘you can do anything in the city and it’s okay’ (Interview, AEJ, 28 April 2023). This limited sense of ownership undermines collective and long-term efforts. Also, as stated by one LCC official using the example of a costly community alarm, worries about vandalism at times hinder implementation efforts (Interview, LCC officer, 22 January 2024).

The previous section illustrated the challenges faced by DRR practitioners working at the city level. Here, it must be noted that significant improvements have recently been made, and that the outlined challenges may differ between actors as well as urban areas. Still, tied to the national DRR policies and their shortcomings in accounting for urban risks and vulnerabilities, these challenges co-create an environment of urban risk accumulation in Lilongwe. The next section provides insights into how urban risk accumulation influences urban living conditions at the ward level.

### Ward level: Insights into the impacts of the urbanisation of risks from Kawale

‘I have stayed in this area for 58 years. But only in the last four to five years, we started having issues with flooding.’ (Interview, Chief Kawale, 05 July 2022)

**FIGURE 4:**
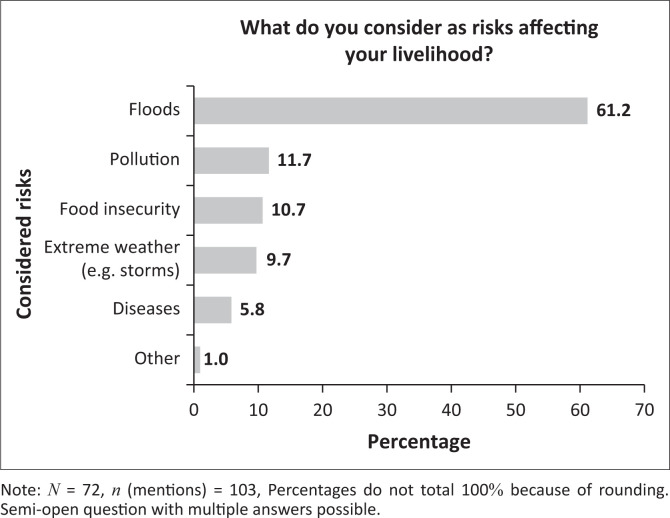
Considered risks, Kawale.

Flooding is the risk most often indicated by respondents in Kawale when asked about risks to their livelihoods (see Figure 4). This is consistent with the municipal communications and the qualitative interviews conducted in the ward. However, given that food insecurity is a perpetual national challenge (GoM 2018), the prioritisation of flooding is still somewhat surprising. In addition to flooding, extreme weather – broadly defined as heavy rains, storms and prolonged dry spells – and pollution are key concerns. Pollution, or waste management, is also a major concern in Kawale, as there is no official dump site. Generally, only 8 percent of solid waste is collected in the city of Lilongwe (UN Habitat 2023:4). Diseases were also considered by a small number of respondents, although one might have expected them to be more frequently mentioned, given the ongoing cholera pandemic since 2022. However, this concurs with findings from Karonga, which showed that preventable diseases were rarely considered as ‘risks’ (UN Habitat 2020:27).

Urban flooding exemplifies the role of the city in the accumulation of risks. Most causes of flooding that are mentioned by the respondents are either urban phenomena or exacerbated in urban environments. Riverbed encroachment and deforestation were linked to the demand for land, housing and fuelwood in Lilongwe. Pollution was also closely related to increased flood risks, pointing at generally flood-prone infrastructure, with solid waste blocking bridges, pipes or drainage systems. The qualitative interviews also highlighted participants’ association of flooding with displacement. Urban dwellers living close to the Lilongwe River often declined to participate in the interviews or questionnaires because of a suspected connection between the researchers and the DoDMA. They feared that the data gathered would ultimately be used as an argument for their displacement. With few housing alternatives, relocation as part of attempts to mitigate flooding was seen to pose a greater risk than the flooding itself. Challenges regarding flood risk management, as well as attempts of (forced) resettlement, were also reflected on by the ward councillors:

‘[*T*]he government does not help in the construction of these people’s houses in towns; DoDMA and the Red Cross only help with foodstuffs […]. However, in the villages, the government helps with construction materials […]. And chiefs are told to move these people […] when floods hit, but these people have nowhere to go.’ (Group Discussion, Kawale, 17 October 2022)

The other ward councillor added:

‘We need some civic education on these people living in these disaster-prone areas because some people are right in the water bodies and helping them after every rainy season is not sustainable. The government should help in giving these people some pieces of land in the upper land […] and help them to move.’ (Group Discussion, Kawale, 17 October 2022)

These comments are supported by complaints voiced by chiefs in rural Malawi of the government delegating relocation activities to the local authorities (Kita 2019). They also hint at differences in support given to rural versus urban areas, and at local authorities being overtasked with actions beyond their actual capacity (Makuwira 2022:57). Relocation is a challenge *per se.* Until the most recent DRM Act, the DoDMA itself had no legal mandate to force the relocation of people settled in declared high-risk areas. And while most respondents from Kawale stressed the need to relocate from flood-prone areas, local authorities’ options are limited to encouraging relocation and issuing civil sanctions, such as refusing emergency support. Yet, these might further increase vulnerability. These challenges also illustrate some of the difficulties of community-based DRR (Dodman & Mitlin 2013).

**FIGURE 5:**
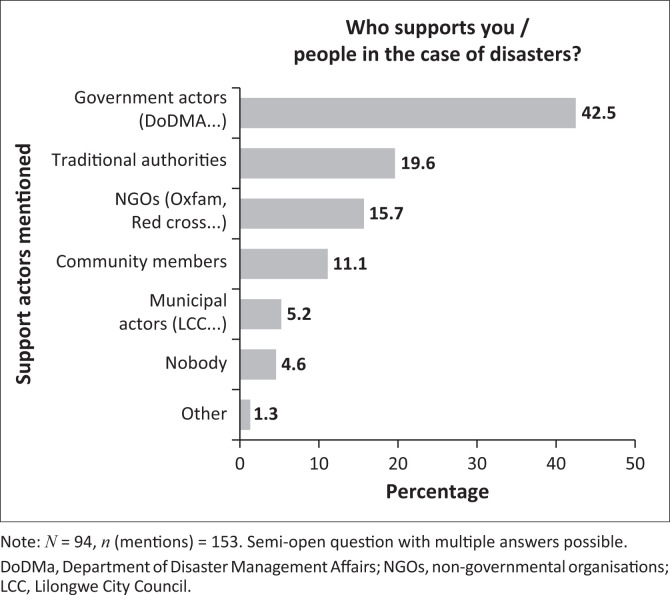
Support actors in the case of disasters, Kawale.

Interestingly, while the block leaders and ward councillors interviewed indicated their extensive role in DRR and post-disaster activities, the questionnaire results point to government actors as being viewed as the most important caretakers (see Figure 5). While this is an encouraging sign given the public scepticism towards the DoDMA, it suggests a mismatch between the burden perceived by local authorities and the (non)recognition of their work by urban residents. This could be explained by the unfeasibility of the tasks delegated to the local authorities or by the limited knowledge urban residents have about civic organisational structures, responsibilities and duties. Furthermore, DoDMA officials usually accompany activities carried out by other organisations, which might lead to the perception that the national agency is in charge. This could also partly explain the limited recognition of NGOs, whose role is vastly below expectations given the donor-based system (Kita 2017a). This finding was supported in part by one chief, who stated that:

‘[*N*]o NGOs are currently providing assistance in emergency situations.’ (Interview, Kawale, 02 July 2022)

The block leaders referred to one active organisation, however, they were highly critical of it, as they perceived its observed results to be negligible compared to the (rumoured) funding it had received (Group Discussion, Kawale, 17 October 2022). Also, the fact that DRR work in urban areas has only recently started is likely to be mirrored in the questionnaire results. Lastly, municipal actors appear to play only a marginal role. This could reflect the limited capacities of the municipalities to respond to disasters or the proximity of DoDMA’s main office on Capital Hill. Yet, it also resonates with a general dissatisfaction with the city council – and with the multiparty democracy – voiced by the ward representatives (Group Discussion, Block Leaders, Kawale, 17 October 2022). However, it is imperative here to point at the limits of representation. Whereas all interviewed block leaders were alive during the pre-democratic era before 1994, the median age of the Malawian population stood at 17 in 2018 (National Statistical Office 2019:10).

## Discussion

### From National neglect to urban riskscapes

This article has traced the urbanisation of risk in Malawi across different scales. At the institutional level, risk remains peripheral in an underfunded, disaster-centric policy framework shaped by recent events. This, combined with a historical neglect of urban areas because of anti-urban policies and scalar mismatches, creates an institutional void facilitating urban risk accumulation. Furthermore, the work of NGOs and governmental actors alike is hampered by a lack of data, limited knowledge of best practice and enforcement issues. As a result, DRR in urban areas is a challenging work in progress, especially considering the uptake of small-scale risks and everyday hazards, and despite the potential brought by new actors entering urban settings and the new DRM bill. For example, in Kawale, unlike the district of Lilongwe, flooding is seen as the main risk. This is partly related to pollution, an everyday hazard in itself, with solid waste blocking pipes, putting further pressure on generally flood-prone infrastructure. Encroachment, deforestation and sand mining were also frequently mentioned as causes of urban flooding. The risks mentioned largely conform with the inventory of risks across the spectrum in Karonga, as presented by Manda and Wanda (2017). This could allow for comparative urban DRR research, while also enabling practitioners to draw from experiences in other cities. However, as illustrated by the difference in flood prevalence between city and district, as well as the relatively low prioritisation of food insecurity at the city level, which is generally a main concern at the national level, research and practices must pay specific attention to risks and vulnerabilities in Malawian cities.

**FIGURE 6:**
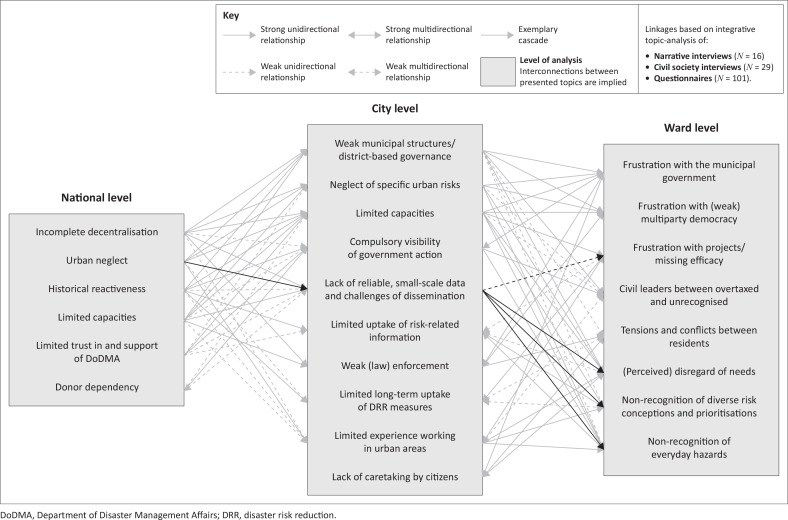
Exemplary illustration of the multilevel challenges facilitating the urbanisation of risk.

The Malawian city must be understood as an arena of conflict, shaping the accumulation of risks, as well as the efficacy of DRR. Mutual accusations of carelessness or a lack of support from the city council and urban residents reveal a glaring mismatch in their respective expectations. The city, maligned by decentralisation, cannot fulfil its mandate to support its citizens. Conversely, a limited sense of ownership on the side of the residents limits the effectiveness of DRR actions taken with limited resources. Furthermore, the most vulnerable, for instance, impoverished urban residents living in informal houses bordering rivers, are at risk of being excluded from the ‘community’ while contending with the paired dangers of floods and displacement. Figure 6 illustrates these challenges of DRR governance for and in urban areas, and how they interrelate at the local level. Of course, relations as depicted here might present differently in urban contexts other than the city of Lilongwe, and present other specific challenges. However, this simplified illustration may act as a starting point to further explore the interplay between policies and actors, and how they address urban risks.

Clearly, there is a need for more research to fully outline the urbanisation of risks in Malawi. While this article details compounding challenges of risk governance at different levels, structural preconditions shaping the accumulation of risks in urban Malawi need to be understood better. Some recent studies provide potential starting points hereto. Tiwale (2019), for example, points at historically inequitable water-infrastructure supply that renders marginalised urban wards water scarce, potentially forcing residents to draw on unsafe water sources, thus further exposing them to neglected everyday hazards. Similarly, apartheid-informed early urban structure plans in Lilongwe City predispose certain population groups to risks of flooding because of locating their settlement areas close to the rivers (Zimba 2022). Gondwe, Manda and Kamlomo (2017) redraw similar patterns of inequitable land allocation and flood risk for Karonga, thereby underlining the need to further interrogate the interplay between inequalities inscribed into the urban form and risks.

Besides patterns of urban risk accumulation, the risk mitigation potential of urban environments in Malawi, as well as specific coping strategies by residents need further unpacking to harness their potential for risk reduction (UN Habitat 2020). For that, in-depth qualitative studies are needed. As a potential starting point, Khumalo (2023) analyses place-making processes and the agency of urban residents settling in disaster-prone areas in Blantyre, thereby sensitising for both place attachment and specific coping mechanisms of urban residents living at risk. Similarly, assessing (informal) civic arrangements to mitigate risks could help to better understand residents’ agency and demands and allow for adapted supportive measures. However, as this study indicated, residents, especially of informal settlements, might be sceptical of government action because of fears of subsequent displacement. Research and recommendations must be aware of these worries to ensure their efficacy and adequacy. On the positive side, sensitive collaborative approaches respectful of lived realities in informal settlements might help to combat the ongoing urbanisation of risks – and to improve living conditions of the majority of the urban population.

## Conclusion

The accumulation of risks in urban areas is multidimensional (Bull-Kamanga et al. 2003; UN Habitat 2020). This article sought to provide entry points to understanding this process in urban Malawi by underlining and illustrating the challenges faced by institutions and actors working at the city level, linking them to (some of) the risks faced by urban residents in Kawale, Lilongwe. Clearly, cities are not inherently resilient, as has long been presumed. Although the recent turn towards the city in the wake of Cyclone Freddy is encouraging, it is imperative for policies to transcend the disaster context and to proactively address risks and vulnerabilities in cities. Of course, this is not to deny the extreme vulnerabilities in rural areas of Malawi or to neglect the efforts made by institutions working with very limited resources. However, given the urban growth trajectory and the illustrated urbanisation of risk, urban DRR must be promoted to mitigate future disasters. Therefore, the implementation of urban DRR methods must understand and consider the city as a specific arena of conflict, risk accumulation and potential. To achieve this, empowering city-level actors, as well as including urban residents and their knowledge, is important.
